# Risk of Nonaccidental and Cardiovascular Mortality in Relation to Long-term Exposure to Low Concentrations of Fine Particulate Matter: A Canadian National-Level Cohort Study

**DOI:** 10.1289/ehp.1104049

**Published:** 2012-02-07

**Authors:** Dan L. Crouse, Paul A. Peters, Aaron van Donkelaar, Mark S. Goldberg, Paul J. Villeneuve, Orly Brion, Saeeda Khan, Dominic Odwa Atari, Michael Jerrett, C. Arden Pope, Michael Brauer, Jeffrey R. Brook, Randall V. Martin, David Stieb, Richard T. Burnett

**Affiliations:** 1 Environmental Health Science and Research Bureau, Health Canada, Ottawa, Ontario, Canada; 2 Health Analysis Division, Statistics Canada, Ottawa, Ontario, Canada; 3 Department of Physics and Atmospheric Science, Dalhousie University, Halifax, Nova Scotia, Canada; 4 Department of Medicine, McGill University, Montreal, Quebec, Canada; 5 Dalla Lana School of Public Health, University of Toronto, Toronto, Ontario, Canada; 6 School of Public Health, University of California–Berkeley, Berkeley, California, USA; 7 Department of Economics, Brigham Young University, Provo, Utah, USA; 8 School of Population and Public Health, University of British Columbia, Vancouver, British Columbia, Canada; 9 Air Quality Research Division, Environment Canada, Downsview, Ontario, Canada; 10 Harvard-Smithsonian Center for Astrophysics, Cambridge, Massachusetts, USA

**Keywords:** Canada, cardiovascular mortality, cohort study, fine particulate matter

## Abstract

Background: Few cohort studies have evaluated the risk of mortality associated with long-term exposure to fine particulate matter [≤ 2.5 μm in aerodynamic diameter (PM_2.5_)]. This is the first national-level cohort study to investigate these risks in Canada.

Objective: We investigated the association between long-term exposure to ambient PM_2.5_ and cardiovascular mortality in nonimmigrant Canadian adults.

Methods: We assigned estimates of exposure to ambient PM_2.5_ derived from satellite observations to a cohort of 2.1 million Canadian adults who in 1991 were among the 20% of the population mandated to provide detailed census data. We identified deaths occurring between 1991 and 2001 through record linkage. We calculated hazard ratios (HRs) and 95% confidence intervals (CIs) adjusted for available individual-level and contextual covariates using both standard Cox proportional survival models and nested, spatial random-effects survival models.

Results: Using standard Cox models, we calculated HRs of 1.15 (95% CI: 1.13, 1.16) from nonaccidental causes and 1.31 (95% CI: 1.27, 1.35) from ischemic heart disease for each 10-μg/m^3^ increase in concentrations of PM_2.5_. Using spatial random-effects models controlling for the same variables, we calculated HRs of 1.10 (95% CI: 1.05, 1.15) and 1.30 (95% CI: 1.18, 1.43), respectively. We found similar associations between nonaccidental mortality and PM_2.5_ based on satellite-derived estimates and ground-based measurements in a subanalysis of subjects in 11 cities.

Conclusions: In this large national cohort of nonimmigrant Canadians, mortality was associated with long-term exposure to PM_2.5_. Associations were observed with exposures to PM_2.5_ at concentrations that were predominantly lower (mean, 8.7 μg/m^3^; interquartile range, 6.2 μg/m^3^) than those reported previously.

Effects on cause-specific mortality from long-term exposure to fine particulate matter [≤ 2.5 μm in aerodynamic diameter (PM_2.5_)] have been investigated in only a handful of cohort studies (Chen et al. 2008). Most notably, two large prospective cohort studies based in the United States, the American Cancer Society (ACS) Cancer Prevention II study (Krewski et al. 2009; Pope et al. 1995, 2002) and the Harvard Six Cities study (Dockery et al. 1993; Laden et al. 2006), showed robust and statistically significant positive associations between long-term exposure to concentrations of ambient pollution and mortality from cardiopulmonary diseases and lung cancer after adjusting for smoking and other risk factors. A systematic review of the association between long-term exposure to ambient pollution and chronic diseases conducted by Chen et al. (2008) concluded that long-term exposure to PM_2.5_ increases the risk of cardiovascular mortality by approximately 12–14% per 10-μg/m^3^ increase in PM_2.5_, independent of age, sex, and geographic region.

Most studies of ambient associations between air pollution and health have relied on observations from relatively sparse networks of ground-based pollution monitors over relatively short periods of time. In Canada, for example, even large cities have had relatively few permanent fixed-site pollution monitors operating over the last two decades, and there are few stations in rural and remote locations of the country. Use of only the available monitors to assign exposure necessitates restricting the population studied to residents living within a certain distance from monitors and/or deriving estimates of exposure at more distant locations through spatial interpolation.

In the present study, we analyzed Canadian national-level cohort data in order to investigate cause-specific risks for mortality associated with long-term exposure to PM_2.5_. First, we present an analysis based on Environment Canada’s network of ground-based pollution monitoring stations in 11 of Canada’s largest cities; this necessitated using only a subset of the cohort for which exposure could reasonably be assigned from the network data. Then, to include the whole cohort, we applied estimates of concentrations of ground-level PM_2.5_ throughout the country from satellite observations of aerosol optical depth (van Donkelaar et al. 2010).

## Methods

*The study cohort.* The study cohort is a subset of the 1991–2001 Canadian census mortality follow-up study (Wilkins et al. 2008). Persons were eligible for the census mortality cohort if they were ≥ 25 years of age; were a usual resident of Canada on the census reference day (4 June 1991); were not a long-term resident of an institution such as a prison, hospital, or nursing home; and had been among the 20% of Canadian households (~ 3.6 million respondents) selected randomly for enumeration with the mandatory long-form questionnaire. Subjects in the census cohort were linked to the Canadian Mortality Database (Statistics Canada 2005b) from 4 June 1991 to 31 December 2001 using deterministic and probabilistic linkage methods (Wilkins et al. 2008). A random selection of 125,100 linked records were excluded from the cohort so that the final sample represented no more than 15% of the Canadian population, as stipulated in the record linkage protocol, which left ~ 2.7 million subjects in the cohort (i.e., ~ 76% of the 3.6 million respondents). The 1991–2001 Canadian census mortality follow-up study received approval by the Statistics Canada Policy Committee (reference no. 012-2001) after consultation with Statistics Canada Confidentiality and Legislation Committee, Data Access and Control Services Division, and the Federal Privacy Commissioner. This approval is equivalent to that of standard research ethics boards.

In the present study, we included only subjects who were nonimmigrants (i.e., only those granted Canadian citizenship by birth, which left ~ 2.1 million subjects for the analysis) because we were interested in capturing the exposure experience of longer-term residents of Canada. Immigrants to Canada have unknown prior exposures and are more likely to live in areas that are characterized by higher ground-level concentrations of PM_2.5_ (e.g., large cities such as Toronto, Montreal, and Vancouver) than those born in Canada (Villeneuve et al. 2011).

Immigrants, and especially recent immigrants, tend to have better health and health behaviors than the Canadian-born population (Ali et al. 2004; McDonald and Kennedy 2004) and to live longer than the nonimmigrant population (Wilkins et al. 2008). Because of more limited information on long-term exposure and because of immigrant-related traits that complicate the analysis, the immigrant subset of the population was excluded from our analysis and will be the subject of a separate, future analysis.

Mortality data included underlying cause of death [coded to the *International Classification of Diseases, 9th Revision* (ICD-9; WHO 1977), for deaths before 2000 and to ICD-10 (WHO 1992) for those deaths registered from 2000 onward] and date of death. Additionally, location of residence of each subject at baseline was aggregated to 1991 enumeration areas. Enumeration areas range in size from approximately 650 dwellings in urban areas to < 100 dwellings in rural areas. In 1991 there were 45,710 enumeration areas across the country.

*Assignment of concentrations of PM_2.5_.* Observations of concentrations of PM_2.5_ from ground-based stations were available for the follow-up period of the cohort in only 11 cities. We calculated the mean annual concentration in these cities averaged over the 1987–2001 period (i.e., 5 years before baseline and the full 10 years of follow-up) and assigned exposure to each cohort member living in the corresponding 11 census divisions [for details regarding the compilation of the historical ground-based observations, see Supplemental Material (http://dx.doi.org/10.1289/ehp.1104049)]. This subsample represented 43% of the full cohort.

A second set of exposure estimates was created for the full cohort using estimates of PM_2.5_ derived from satellite remote sensing observations during the period 2001–2006 [van Donkelaar et al. 2010; for additional descriptions of the satellite data retrievals, see Supplemental Material (http://dx.doi.org/10.1289/ehp.1104049)]. These estimates were available on a grid with a spatial resolution of 10 km × 10 km. Previous analyses showed that the satellite-derived estimates of PM_2.5_ were in close agreement (Pearson correlation coefficient *r* = 0.77, slope = 1.07, *n* = 1,057) with ground-based measurements both in Canada and in the United States (van Donkelaar et al. 2010). We overlaid the surface on a map layer representing the 1991 enumeration area boundaries and computed the mean concentration of PM_2.5_ within the boundaries of each enumeration area across the country. We thus assigned exposure to the satellite-derived estimates of PM_2.5_ to all cohort members by linking the exposure surface to their enumeration area of residence in 1991. The sparsely inhabited northern territories of Canada were excluded from our study because of the absence of estimates of PM_2.5_.

*Contextual variables.* Geographical context can influence risk of mortality and may confound the association between mortality and air pollution because areas characterized by some population groups (e.g., those of lower income, the unemployed) and by some environmental characteristics (urban vs. rural) tend also to be characterized by higher ambient concentrations of air pollution (Crouse et al. 2009; Jerrett et al. 2001). To derive ecological covariates, we aggregated census data from 1991 describing socioeconomic and demographic characteristics to the smaller “neighborhood” scale of census tracts as well as to the larger “community” scale of census divisions, which may correspond to (or be of comparable size to) counties. To adjust for regional variations in these variables across Canada, we subtracted the census division mean from the values of each census tract. We thus compiled ecological variables describing the proportion of unemployed adults (≥ 15 years of age), the proportion of adults who had not completed high school, and the proportion of individuals in the lowest income quintile (as represented by the ratio of family income divided by the low-income cutoff) at both scales for each cohort member. The low-income cutoff, which varies by community and family size, is defined by Statistics Canada to identify those who need to spend a greater proportion of their income on basic necessities than does an average family of similar size and thus provides an indicator of deprivation that is adjusted for regional variation in the cost of living (Statistics Canada 2001). We also created a categorical variable that indicated the population size of the subject’s home community given that those who live in rural areas tend to have poorer health than those who live in more urban areas (Eberhardt and Pamuk 2004).

*Statistical methods.* We estimated hazard ratios (HRs) using a standard Cox proportional hazards model as well as with a nested, spatial random-effects Cox model (Krewski et al. 2009; Ma et al. 2003), in which random effects were represented by clusters, as defined below. The baseline hazard function for both models was stratified by single-year age groups and sex. Follow-up time was measured in days, calculated from 4 June 1991 to 31 December 2001.

The standard Cox survival model assumes that the survival time of each subject is statistically independent from that of other subjects after controlling for the mortality risk factors included in the model. These risk factors, however, may not completely explain differences in mortality between subjects and may introduce dependencies among subjects. One approach to explain some of these dependencies is to include information on the location of each subject, such as their community and neighborhood. Subjects living in the same community or neighborhood may be more likely to share mortality risk factors than subjects residing in different locations.

In our nested, spatial random-effects Cox model, therefore, we defined two levels of spatial clusters: a first cluster level defined by census divisions, and a second cluster level defined by census tracts within census divisions. We assumed that if two census divisions were adjacent, then they were correlated, and if not adjacent, uncorrelated. A similar assumption was made for the census tracts within each census division. Census tracts in different census divisions were assumed to be uncorrelated.

We examined the sensitivity of the estimates of the effects of air pollution on mortality due to the proportional hazard assumption using methods presented by Sheppard et al. (2011). We developed survival models for each year of follow-up and summarized the HRs for PM_2.5_ over the multiple years, and we found that the HR and its associated standard error was similar to those achieved by a single, multiyear model, which suggested that our estimate of the PM_2.5_ HR and the corresponding standard error was not sensitive to the proportional hazards assumption.

We also examined the sensitivity of the PM_2.5_ association with mortality to inclusion of selected sets of covariates: those measured at the subject level (i.e., those covariates listed in Table 1), an indictor of urban size, and the contextual covariates measured at the census division and census tract levels.

**Table 1:** Descriptive statistics for the study cohort.

Variable	Subjects [n (%)]a	PM2.5 exposure (mean ± SD)
Full cohort		2,145,400 (100)		8.7 ± 3.9
Sex				
Male		1,059,400 (49)		8.6 ± 3.9
Female		1,086,000 (51)		8.7 ± 3.9
Age at entry (years)				
25–34		655,220 (30)		8.7 ± 4.0
35–44		566,900 (26)		8.5 ± 3.8
45–54		349,800 (16)		8.5 ± 3.8
55–69		374,100 (17)		8.8 ± 4.0
≥ 70		202,400 (9)		8.8 ± 4.0
Any aboriginal ancestry				
No		2,047,500 (95)		8.8 ± 3.9
Yes		97,900 (5)		6.3 ± 3.3
Visible minority				
No		2,124,600 (99)		8.6 ± 3.9
Yes		20,800 (1)		10.0 ± 4.7
Marital status				
Married/common-law		1,572,900 (73)		8.5 ± 3.8
Divorced/separated/widowed		285,700 (13)		8.9 ± 4.0
Single		286,800 (13)		9.4 ± 4.2
Highest level of education				
< High school graduation		747,700 (35)		8.2 ± 3.8
High school graduation with or without trade certificate		793,500 (37)		8.6 ± 3.9
Some postsecondary, or college diploma		334,000 (16)		8.9 ± 3.9
≥ University degree		270,200 (13)		9.7 ± 4.2
Employment status				
Employed		1,412,500 (66)		8.8 ± 3.9
Unemployed		130,800 (6)		8.0 ± 3.9
Not in the labor force		602,100 (28)		8.5 ± 3.9
Occupational classification				
Management		174,600 (8)		9.1 ± 4.0
Professional		240,000 (11)		9.2 ± 4.1
Technical		521,600 (24)		8.4 ± 3.8
Semiskilled		528,700 (25)		8.7 ± 3.9
Unskilled		161,500 (8)		8.2 ± 3.8
Not applicable		519,000 (24)		8.6 ± 3.9
Low-income cutoff quintile				
Lowest		470,700 (22)		8.4 ± 3.8
Lower middle		450,300 (21)		8.5 ± 3.8
Middle		377,500 (18)		8.7 ± 3.9
Upper middle		437,900 (20)		8.8 ± 3.9
Upper		409,100 (19)		9.0 ± 4.1
Size of home community (population)				
Rural/farm		585,900 (27)		6.5 ± 2.6
Small town (< 30,000)		326,400 (15)		6.6 ± 2.8
Urban 3 (30,000–99,999)		216,200 (10)		7.6 ± 2.7
Urban 2 (100,000–499,999)		233,600 (11)		9.4 ± 4.1
Urban 1 (> 500,000)		783,400 (37)		11.1 ± 3.8
aRounded to nearest hundred to meet the confidentiality restrictions of Statistics Canada.				

We examined the shape of the relationship between PM_2.5_ and mortality using natural cubic spine functions with one, two, three, or four degrees of freedom (df) for both the standard Cox survival model and the spatial random-effects model, and for several causes of death. We then examined plots of the concentration–response curves and used the Bayesian information criterion (BIC) to assess the relative goodness of fit for these models.

We developed models for mortality from ischemic heart disease (ICD-9: codes 410–414; ICD-10: I20–I25), cerebrovascular disease (ICD-9: 430–434, 436–438; ICD-10: I60–I69), cardiovascular disease (ICD-9: 410–417, 420–438, 440–449; ICD-10: I20–I28, I30–I52, I60–I79), circulatory disease (ICD-9: 390–459; ICD-10: I00–I99), and all nonaccidental causes (ICD-9 codes < 800; ICD-10 codes starting with letters A through R). HRs and 95% confidence intervals (CIs) were calculated for an increment of 10 μg/m^3^ in estimated concentrations of PM_2.5_.

## Results

**Figure 1:**
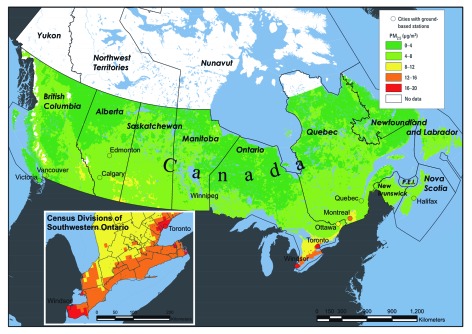
Mean satellite-derived estimates of PM_2.5_ across Canada, 2001–2006, and the mean concentrations in the 11 cities included in our subcohort analysis. P.E.I., Prince Edward Island.

Of the 2.1 million subjects, 49% were men (Table 1), and the mean age at baseline was 45.3 years. There were ~ 21.7 million person-years of follow-up in the cohort and nearly 200,000 nonaccidental deaths. Generally, concentrations of PM_2.5_ were highest in urban areas, along the corridor between Windsor and Quebec City, and in the prairies of southern Saskatchewan and Alberta (Figure 1). Among all subjects, the minimum concentration was 1.9 μg/m^3^, the median was 7.4 μg/m^3^, the mean was 8.7 μg/m^3^, the maximum was 19.2 μg/m^3^, and the interquartile range was 6.2 μg/m^3^. The three ecological covariates (i.e., percent adults without a high school diploma, percent individuals in the lowest income quintile, percent unemployed adults) were negatively correlated with PM_2.5_ (Table 2).

**Table 2:** Descriptive statistics for the ecological covariates.

					Correlation with mean PM2.5
Percentile					
Variable	Minimum	5th	25th	50th		75th	95th	Maximum
Summarized by census tract												
Percent adults without a high school diploma		0.0		17.2	28.8	37.6	45.7	57.1		100		–0.09
Percent individuals in the lowest income quintile		0.0		6.8	12.5	18.0	24.7	39.5		100		–0.03
Percent unemployed adults		0.0		4.3	6.3	8.0	10.0	14.5		66.7		–0.12
Summarized by census division												
Percent adults without a high school diploma		23.7		27.2	32.0	36.3	41.8	53.3		72.9		–0.13
Percent individuals in the lowest income quintile		5.9		11.6	17.8	20.6	21.7	26.8		45.3		–0.06
Percent unemployed adults		2.9		5.9	7.4	8.1	9.2	13.2		26.8		–0.15


*Subcohort analysis.* Among the 11 Canadian cities for which ground-based observations were available, we found very similar patterns (i.e., *r* = 0.84) of concentrations of PM_2.5_ when comparing the 2001–2006 remote sensing-based observations (mean, 9.4 μg/m^3^) with the 2001–2006 ground-based observations (mean, 8.9 μg/m^3^) and with the 1987–2001 ground-based observations (mean, 11.2 μg/m^3^; *r* = 0.89). We found almost identical associations for nonaccidental mortality in this subgroup of the cohort using the standard Cox model (adjusted only for individual-level covariates) with ground-based observations (HR for an increase of 10 μg/m^3^ = 1.11; 95% CI: 1.07, 1.15) and with satellite-derived estimates (HR 1.11; 95% CI = 1.09, 1.13). These latter results included subjects in only 11 cities and were very similar to the estimate for the entire cohort (standard Cox, individual-level covariates only, HR = 1.13; 95% CI: 1.12, 1.14).

**Figure 2:**
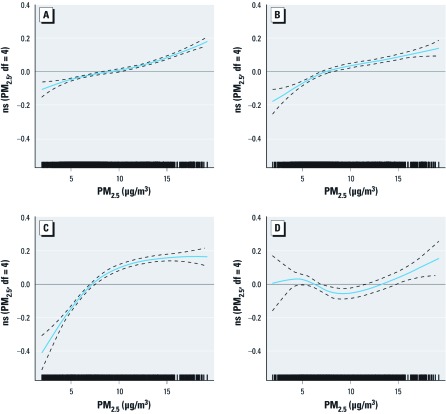
Concentration–response curves (solid lines) and 95% CIs (dashed lines) based on natural spline (ns) models with 4 df, standard Cox models stratified by age and sex, adjusted for all individual-level covariates, urban/rural indicator, and ecological covariates. (*A*) Nonaccidental causes. (*B*) Cardiovascular disease. (*C*) Ischemic heart disease. (*D*) Cerebrovascular disease. The tick marks on the *x*-axis identify the location of the PM_2.5_ concentrations.

*Main findings.* We present in Table 3 the HRs and 95% CIs (from standard Cox and from spatial random-effects models adjusted for personal and contextual covariates) for the associations between PM_2.5_ and selected cardiovascular causes of death among subjects in the full cohort. The HR estimate for all nonaccidental causes from the fully adjusted standard Cox model was 1.15 (95% CI: 1.13, 1.16), and the corresponding HR from the random-effects model was 1.10 (95% CI: 1.05, 1.15). We estimated the strongest association with ischemic heart disease: the HR from the fully adjusted standard Cox model was 1.31 (95% CI: 1.27, 1.35), and the HR based on the random-effects model was 1.30 (95% CI: 1.18, 1.43). We estimated positive and almost identical associations with mortality from cardiovascular and circulatory disease using both model structures. We found no evidence of a linear association with cerebrovascular disease (standard Cox model: HR = 1.04; 95% CI: 0.99, 1.10; random-effects model: HR = 1.04; 95% CI: 0.93, 1.16). This lack of association for cerebrovascular mortality was also supported by the natural spline representation, which did not display a clear increasing mortality risk with PM_2.5_ concentrations (Figure 2D).

**Table 3:** HRs (95% CIs) in the full cohort for an increase of 10 μg/m3 in PM2.5 (satellite-derived estimates) by cause of death and model specification.

Cause of death/model	na	PM2.5 only	PM2.5 covariates	PM2.5 covariates + ecological covariates	PM2.5 covariates + urban/rural indicator	PM2.5 covariates + ecological covariates + urban/rural indicator
Nonaccidental		192,300										
Cox model				1.07 (1.06, 1.08)		1.13 (1.12, 1.14)		1.11 (1.10, 1.12)		1.14 (1.12, 1.16)		1.15 (1.13, 1.16)
Random effects				1.06 (1.01, 1.11)		1.12 (1.07, 1.17)		1.06 (1.01, 1.10)		1.12 (1.07, 1.18)		1.10 (1.05, 1.15)
Cardiovascular		72,600										
Cox model				1.04 (1.02, 1.06)		1.10 (1.08, 1.12)		1.09 (1.07, 1.11)		1.16 (1.13, 1.18)		1.16 (1.13, 1.19)
Random effects				1.05 (0.99, 1.12)		1.11 (1.03, 1.18)		1.07 (1.00, 1.15)		1.17 (1.09, 1.26)		1.15 (1.07, 1.24)
Circulatory		74,700										
Cox model				1.04 (1.02, 1.06)		1.10 (1.08, 1.12)		1.09 (1.07, 1.11)		1.15 (1.13, 1.18)		1.16 (1.13, 1.18)
Random effects				1.04 (0.98, 1.11)		1.10 (1.03, 1.17)		1.06 (0.99, 1.14)		1.16 (1.08, 1.25)		1.14 (1.06, 1.22)
Ischemic heart disease		43,400										
Cox model				1.14 (1.12, 1.17)		1.21 (1.19, 1.24)		1.22 (1.19, 1.25)		1.31 (1.27, 1.34)		1.31 (1.27, 1.35)
Random effects				1.16 (1.06, 1.27)		1.22 (1.11, 1.33)		1.18 (1.08, 1.29)		1.32 (1.20, 1.45)		1.30 (1.18, 1.43)
Cerebrovascular		13,300										
Cox model				0.94 (0.90, 0.99)		0.99 (0.95, 1.03)		0.97 (0.93, 1.02)		1.05 (0.99, 1.10)		1.04 (0.99, 1.10)
Random effects				0.95 (0.87, 1.04)		1.01 (0.91, 1.13)		0.96 (0.87, 1.07)		1.08 (0.96, 1.21)		1.04 (0.93, 1.16)
All models are stratified by age and sex. aRounded to nearest hundred to meet the confidentiality restrictions of Statistics Canada.												

Modeling PM_2.5_ using natural splines did not improve model fit (based on BIC) relative to models that assumed linearity for nonaccidental, cardiovascular, or cerebrovascular deaths (Figure 2A,B,D). However, using a natural spline model with 4 df yielded lower BIC than other alternatives for ischemic heart disease mortality (Figure 2C).

Following Krewski et al. (2009), who reported that the logarithmic function of PM_2.5_ was a better predictor for mortality from ischemic heart disease than was the linear model in analyses of the ACS cohort, and based on the shape of the concentration–response curve (see Figure 2C), we fitted a model with ln(PM_2.5_ + 1). This model specification yielded a lower BIC than each of the spline representations. The mean ± SE coefficient for our logarithm model for ischemic heart disease was 0.3031 ± 0.0152 for the standard Cox survival model and 0.2894 ± 0.0440 for the random-effects survival model. This logarithmic model predicted a relative risk in ischemic heart disease mortality of 1.20 associated with a change in PM_2.5_ exposure from 5 μg/m^3^ to 10 μg/m^3^ and a relative risk of 1.12 based on the change in exposure from 10 μg/m^3^ to 15 μg/m^3^.

## Discussion

We found positive and statistically significant associations between nonaccidental mortality and estimates of PM_2.5_ generated from both satellite-derived and ground-based observations in a large cohort of nonimmigrant Canadians ≥ 25 years of age. The cohort included subjects from each province and from every major city, as well as those living in rural locations. In addition to adjusting for multiple individual, contextual, and spatial effects, a key strength of this study includes the large sample size and number of deaths necessary to detect mortality associations at relatively low concentrations of PM_2.5_. Our estimated mean concentration of PM_2.5_ for subjects across Canada was 8.7 μg/m^3^, which is substantially lower than the corresponding estimate for subjects across the United States as reported in the ACS study [mean concentration of 14.4 μg/m^3^ (Krewski et al. 2009)]. As reflected in the concentration–response curves presented in Figure 2A,B, we found near linear associations between PM_2.5_ and mortality from nonaccidental and cardiovascular disease. Generally, these plots suggest that associations with mortality were present at concentrations of PM_2.5_ of only a few micrograms per cubic meter.

The ACS study (Krewski et al. 2009; Pope et al. 2002) is similar to the present study in that both considered associations between mortality and PM_2.5_, covered their respective countries, and included both men and women. The HRs for risk of cardiovascular mortality associated with exposure to PM_2.5_ in our cohort are similar to those reported in the ACS study: HRs of ~ 1.10–1.15 for all cardiovascular deaths, stronger associations with ischemic heart disease, and little-to-no association with cerebrovascular mortality (Pope et al. 2004). In the present study, HRs based on the fully adjusted (i.e., individual-level covariates, urban/rural indicator, and ecological covariates) spatial random-effects model were consistent, although slightly attenuated, compared with those based on the standard Cox model (Table 3), whereas in the ACS cohort (see Krewski et al. 2009, their table 9), HRs based on spatial random-effects models tended to be slightly larger than those based on standard Cox models. In the ACS cohort, adjustment for ecological covariates increased the HRs (see Krewski et al. 2009, their table 6), whereas they had little impact in the present study (Table 3). Overall, even though there is some overlap in estimates of risk between this and the ACS cohort, our results emphasize the importance of conducting air pollution studies in regions specifically for which inferences are to be made.

One of us (R.T.B.) identified seven cohort studies that estimated the association between exposure to PM_2.5_ and mortality due to ischemic heart disease (data not shown). The relative risks and 95% CIs for a 10-μg/m^3^ change in ambient PM_2.5_ concentration from these studies were as follows: ACS study (Krewski et al. 2009), 1.29 (1.18, 1.41); Six City Study (Laden et al. 2006), 1.26 (1.08, 1.47); California Teachers Study (Lipsett et al. 2011), 1.21 (1.05, 1.42); Nurses Health Study (Puett et al. 2009), 2.02 (1.07, 3.78); Women’s Health Initiative (Miller et al. 2007), 2.12 (1.17, 4.16); Adventist Study of Health and Smog (Chen et al. 2005), 1.00 (0.87, 1.15); and Netherlands Study of Cancer and Diet (Beelen et al. 2008), 0.96 (0.75, 1.22). One of us (R.T.B.) estimated the meta-analytic uncertainty distribution of these estimates using the *Q*-statistic (Huedo-Medina et al. 2006) and found that the mean of the uncertainty distribution was 1.20 with a 90% coverage interval of 0.98–1.48 (data not shown). Our estimated HR of 1.30 for ischemic heart disease is well within this uncertainty distribution. Furthermore, two expert judgment assessments of the relationship between long-term exposure to PM_2.5_ and mortality (Cooke et al. 2007; U.S. Environmental Protection Agency 2006) estimated HRs for the United States and Europe of approximately 1.10 for all-cause mortality associated with an increase in PM_2.5_ of 10 μg/m^3^, a value identical to the HR that we report here for nonaccidental mortality.

*Strengths of this study.* This is one of the largest cohort studies, and the first national-level Canadian study, to evaluate the risk of mortality associated with long-term exposure to PM_2.5_. The use of remote sensing to estimate ground-level concentrations of PM_2.5_ allowed us to assign an estimate of exposure to virtually all cohort members (excluding those living in the Arctic territories); we were thus not restricted to those living in urban areas and/or within a given distance of a monitoring site. We had information on many important predictors of mortality at both the individual and ecological levels.

The spatial pattern of mortality among census tracts within each census division and among census divisions was captured using our random-effects survival model. This pattern was due to the inability of the covariates in the standard Cox model to explain all the differences in mortality rates across Canada. The estimates of the PM_2.5_ HRs were less sensitive to covariate adjustment in the spatial random-effects survival model compared with the standard Cox model because of the simultaneous modeling of the spatial pattern of mortality, which was not captured by the covariates. The standard Cox model yielded smaller estimates of the standard error of the PM_2.5_ coefficient compared with the spatial random-effects model, suggesting that there was unexplained spatial variation in mortality within the cohort. This result is reflected in the wider CIs around the random-effects HRs. The addition of both the census tract subcluster random-effect and the spatial autocorrelation among clusters and subclusters increased the estimate of the standard error of the PM_2.5_ association with mortality. We concluded that the structure of survival in this cohort that could not be explained by the risk factors included in the model is highly complex, and thus the extension of the Cox survival model is required to capture this complexity more accurately so that risk estimates and CIs are characterized appropriately.

Given that we were interested in chronic exposure to pollution and its relationship to mortality, we sought to include subjects in the cohort who might best represent the long-term Canadian exposure experience. We therefore excluded all immigrants from the cohort. None of the previous cohort studies on air pollution and mortality have considered this issue.

*Limitations of this study.* Mobility of subjects in the cohort is a potential source of exposure misclassification. We knew the home address of subjects in 1991 but did not know their addresses during the follow-up period. Subjects may have moved to locations characterized by lower, higher, or similar levels of pollution. There is an equal likelihood among all subjects of being assigned an inaccurate estimate (either an overestimate or underestimate) of true exposure, which means that our findings may be subject to nondifferential misclassification bias. It is thus likely that our results underestimate the true associations between mortality and PM_2.5_ in this cohort.

We cannot be certain that we have eliminated all sources of residual or unobserved confounding but suspect that any remaining confounding is likely to have only minimal effect on our results. In their analysis of approximately 500,000 adults in the ACS cohort, Pope et al. (2002) reported that relative risk estimates of air-pollution–related mortality were not sensitive to adjustment for body mass index, alcohol consumption, occupational exposures, or diet-related variables (see Pope et al. 2002, their Figure 3).

We lacked information on two of the most important risk factors for cardiovascular disease: smoking and obesity. Villeneuve et al. (2011) examined the association between several mortality risk factors and our estimates of PM_2.5_ exposure by linking these estimates to each subject’s home address postal code in the 2001 panel of the Canadian Community Health Survey (Statistics Canada 2005a). In particular, Villeneuve et al. (2011) found an inverse association between the prevalence of former or current smokers and PM_2.5_ exposures and selected categories of body mass index. This inverse association is consistent with the fact that individuals of higher socioeconomic status tended to live in areas with higher levels of pollution, such as southwestern Ontario and Quebec (data not shown). We found that our HRs associated with exposure to PM_2.5_ increased after adjusting for available individual-level socioeconomic variables. These socioeconomic variables have also been shown to be inversely related to smoking habits and obesity (Villeneuve et al. 2011). We therefore hypothesize that additional statistical adjustment for smoking and body mass index, if available, would increase the PM_2.5_ HR. Although we had no information on diet or alcohol use, we expect that they would be correlated with other indicators of socioeconomic status. Thus, by not adjusting for diet and alcohol, we are likely underestimating the PM_2.5_ risk.

Although using the satellite-derived surface allowed us to estimate exposure to subjects across Canada, the spatial resolution of the surface (i.e., 10 km × 10 km) meant that we were unable to detect spatial variability at finer spatial scales. We acknowledge also that this surface describes only patterns of PM_2.5_ generally and that our analyses do not consider qualitative characteristics of the potential mixture of pollutants to which subjects may have been exposed.

As noted earlier, deaths registered in this cohort before 2000 were coded to ICD-9, and those registered in 2000 and onward were coded to ICD-10. This difference in coding does not introduce risk of misclassification of deaths. We included in our analyses general categories of mortality that have been identified as not having problems with interpretation, and which have comparability ratios (between ICD-9 and ICD-10) close to 1 (see Kochanek et al. 2001, their table II in technical notes).

## Conclusion

We identified in this national-level, population-based cohort study of nonimmigrant Canadians associations between cardiovascular mortality and long-term exposure to PM_2.5_. We found the strongest association with ischemic heart disease, and our HRs were similar in magnitude to those reported in large cohort studies conducted elsewhere. Our associations were reported with exposure to concentrations of PM_2.5_ as low as only a few micrograms per cubic meter.

## Supplemental Material

(106 KB) PDF
