# Supplementary material for: Risk of Nonaccidental and Cardiovascular Mortality in Relation to Long-term Exposure to Low Concentrations of Fine Particulate Matter: A Canadian National-Level Cohort Study
Source: Environ Health Perspect. 2012 Feb 7;120(5):708–14. doi: 10.1289/ehp.1104049 (PMC3346774; doi:10.1289/ehp.1104049)
Supplement: (106 KB) PDF [file ehp.1104049.s001.pdf]

Supplemental Material

Risk of Non-accidental and Cardiovascular Mortality in Relation to Long-term Exposure to Low Concentrations of Fine Particulate Matter: A Canadian National-level Cohort Study

Dan L. Crouse[1], Paul A. Peters[2], Aaron van Donkelaar[3], Mark S. Goldberg[4], Paul J.Villeneuve[1, 5], Orly Brion[1], Saeeda Khan[2], Dominic Odwa Atari[2], Michael Jerrett[6], C. Arden Pope III[7], Michael Brauer[8], Jeffrey R. Brook[9, 5], Randall V. Martin[3, 10], David Stieb[1], Richard T. Burnett[1]

[1]Health Canada, Ottawa, Canada

[2]Statistics Canada, Ottawa, Canada

[3]Dalhousie University, Halifax, Canada

[4]McGill University, Montreal, Canada

[5]University of Toronto, Toronto, Canada

[6]University of California, Berkeley, USA

[7]Brigham Young University, Provo, USA

[8]University of British Columbia, Vancouver, Canada

[9]Environment Canada, Downsview, Canada

[10]Harvard-Smithsonian Center for Astrophysics, Cambridge, USA

Compiling Historical Observations of $PM_{2.5}$

We used mean observations of $PM_{2.5}$ from ground-based stations at 12 locations across Canada (Figure 1). A six-day sampling schedule was used for each station. The 12 locations were chosen because they were the only locations for which data were available for the15-year period including the five years prior to baseline and our ten years of follow-up (i.e., 1987-2001). There were many individual months for which no data were available at some of stations and so to overcome this, we pooled data from stations that were within six kilometres of each other and then calculated monthly, seasonal, annual, and five-year means at each location according to the following criteria:

- There had to be observations for at least three days in a given month for that month to be included
- We divided years into two six-month "seasons", namely cold (i.e., January, February, March, October, November, December) and warm (i.e., April, May, June, July, August, September) and there had to be observations in least three months for a season to be included
- There had to be observations in both seasons for a year to be included (thus, there had to be observations in at least three cold months and at least three warm months)
- We then calculated the mean concentrations for three separate 5-year periods: 1987-1991; 1992-1996; 1997-2001 in order to ensure some data representation over the entire period of interest.

Description of the Satellite-based $PM_{2.5}$ Estimates

We used concentrations of ground-level fine particulate matter ($PM_{2.5}$) inferred from satellite observations of aerosol optical depth (AOD) by van Donkelaar et al. (2010). The AOD data were from the Moderate Resolution Imaging Spectroradiometer (MODIS; Levy et al. 2007) and the Multiangle Imaging Spectroradiometer (MISR; Kahn et al., 2009) satellite instruments. These instruments are located onboard the Terra satellite, which has been circumnavigating the earth approximately 15 times per day since the December 1999. AOD is a measure of the extinction of light by aerosol scattering and absorption in the atmospheric column between the instrument and the surface of the earth.

van Donkelaar et al. (2010) combined the MODIS and MISR AOD with coincident simulated aerosol vertical structure and scattering properties from a chemical transport model (GEOS-Chem; Bey et al., 2001; www.geos-chem.org) to generate a global raster surface (i.e., grid) of $PM_{2.5}$ at a spatial resolution of 0.1° × 0.1°, or approximately 10 km × 10 km at mid-latitudes. Estimates of $PM_{2.5}$ for each grid cell are based on at least 50 satellite retrievals averaged over the six-year period between 2001 and 2006 and corrected for sampling bias. The population-weighted average uncertainty for Canada is 2.5 $\mu g/m^3$. Areas characterised by substantial seasonal variation in AOD, or persistent cloud or snow cover have greater uncertainty. Remote sensing derived $PM_{2.5}$ values were in close agreement (r=0.77, slope=1.07, n=1057) with *in situ* ground-based measurements in both Canada and the United States (van Donkelaar et al. 2010).

Supplemental Material References

Bey, I., D.J. Jacob, R.M. Yantosca, J.A. Logan, B.D. Field, A.M. Fiore, Q. Li, H.Y. Liu, L.J. Mickley, and M.G. Schultz, Global modeling of tropospheric chemistry with assimilated meteorology: Model description and evaluation, *J. Geophys. Res.*, *106*, 23073-23096, 2001.

Kahn RA, Nelson DL, Garay MJ, Levy RC, Bull MA, Diner DJ, et al. 2009. MISR Aerosol Product Attributes and Statistical Comparisons with MODIS. IEEE Transactions on Geoscience and Remote Sensing 47(12): 4095 - 4114.

Levy, R.C., L.A. Remer, S. Mattoo, E.F. Vermote, and Y.J. Kaufman, Second-generation operational algorithm: Retrieval of aerosol properties over land from inversion of Moderate Resolution Imaging Spectroradiometer spectral reflectance, *J. Geophys. Res.*, *112*, D13211, doi: 10.1029/2006JD007811, 2007.

van Donkelaar, A., R.V. Martin, M. Brauer, R. Kahn, R. Levy, C. Verduzco, and P.J. Villeneuve, Global estimates of ambient fine particulate matter concentrations from satellite-based aerosol optical depth: Development and application, *Environ. Health Perspect.*, doi:10.1289/ehp.0901623, 2010.
